# Effect of simvastatin versus calcium hydroxide intracanal medications on postoperative pain and cytokine modulation in symptomatic apical periodontitis: a randomized controlled trial

**DOI:** 10.1186/s12903-026-09793-3

**Published:** 2026-09-15

**Authors:** Mohammed Essam Othman Alghidany, Dina Ahmed Morsy, Olfat Gamil Shaker, Randa Mohamed El Boghdadi

**Affiliations:** 1 Department of Endodontics, Faculty of Dentistry, Cairo University, Cairo, Egypt; 2 Department of Medical Biochemistry and Molecular Biology, Faculty of Medicine, Cairo University, Cairo, Egypt

**Keywords:** Calcium hydroxide, Cytokine, Interleukin-6, Interleukin-8, Intracanal medication, Periapical periodontitis, Postoperative pain, Simvastatin

## Abstract

**Background:**

Calcium hydroxide has long been used as an intracanal medicament in endodontics and remains widely applied due to its well-established antimicrobial and anti-inflammatory properties. Owing to its clinical effectiveness, it is recommended for use in a variety of endodontic treatment situations. Simvastatin, a drug belonging to the statin class, has demonstrated potential immunomodulatory effects on periapical tissues when administered both locally and systemically. This study aimed to assess the effect of using Simvastatin versus calcium hydroxide Ca(OH)₂ intracanal medicaments on incidence and intensity of postoperative pain and periapical fluid levels of Interleukin-6 (IL-6) and Interleukin-8 (IL-8) one week post-instrumentation in patients with necrotic mandibular premolars with symptomatic apical periodontitis.

**Methods:**

Thirty patients diagnosed with single-canaled necrotic premolars, allocated randomly into two groups (*n* = 15 each). Root canal therapy was executed across two clinical sessions. Preoperative pain was recorded using the Numeric Rating Scale (NRS). After chemo-mechanical preparation, post-instrumentation periapical fluid (PS-1) was collected. The intervention group received Simvastatin gel, while the control group received Ca(OH)₂ intracanal medication. Participants self-reported pain intensity at 6-, 12-, 24-, and 48-hour intervals post-instrumentation. After one week, a second periapical sample (PS-2) was collected, and both IL-6 and IL-8 levels were quantified using enzyme-linked immunosorbent assay (ELISA). Final obturation was performed using a modified single-cone techniques with bioceramic sealer. Post-obturation pain was similarly documented. Data were statistically analyzed using Mann-Whitney U test for independent samples comparison, Wilcoxon signed rank test for related samples comparison, Friedman test for multiple related samples comparisons followed by Dunn’s post hoc test for pairwise comparison, and Chi square test was used for independent group comparison of categorical data.

**Results:**

Simvastatin was associated with significantly lower postoperative pain intensity at 6 h post-instrumentation compared to Ca(OH)₂ (*p* = 0.037), while no significant differences were observed at other time intervals. The proportion of patients experiencing postoperative pain did not differ significantly between the two groups. Both medications reduced cytokine levels from baseline; however, intergroup comparisons showed no significant differences. Notably, Simvastatin significantly decreased both IL-6 (*p* = 0.017) and IL-8 (*p* = 0.002) levels, whereas Ca(OH)₂ significantly reduced only IL-8 (*p* = 0.005).

**Conclusions:**

Simvastatin demonstrated comparable efficacy to Ca(OH)₂ in managing postoperative pain and reducing inflammatory mediators, suggesting its potential as an alternative intracanal medication. While Ca(OH)₂ remains the gold standard intracanal medicament, simvastatin demonstrated promising anti-inflammatory effects that may contribute to reduced postoperative pain and decreased levels of pro-inflammatory mediators. Future investigations incorporating larger sample sizes, extended follow-up durations, different simvastatin concentrations, alternative delivery vehicles, long-term radiographic healing outcomes, effect on other inflammatory mediators, and its applicability in different types of apical periodontitis or retreatment cases are essential to corroborate these outcomes and evaluate the expanded clinical utility of simvastatin within endodontic therapy.

**Trial registration:**

The trial was registered at clinicaltrials.gov with Registration number: NCT 05525013. (CEBD-CU-2022-8-30)

## Background

One of the most frequent side effects of endodontic therapy is postoperative pain., occurring in 3% to 58% of cases [1]. It primarily results from inflammation in the periapical tissues (apical periodontitis), which stimulates pain receptors through the release of inflammatory mediators like prostaglandins, leukotrienes, bradykinin, and serotonin [2]. Apical periodontitis is a dynamic process that can last from minutes to years, depending on the injury’s severity and tissue vascularity. The inflammatory response serves to neutralize harmful agents, eliminate dead tissue, and initiate healing. This process entails a dynamic interplay of multifaceted mechanisms, such as hemodynamic alterations, enhanced vascular permeability, immune cell recruitment and activation, and the secretion of soluble signalling molecules. The transition from acute inflammation to resolution is not passive but rather an orchestrated biological process mediated by the synthesis and release of specialized pro-resolving mediators (SPMs) and anti-inflammatory cytokines [3].

The pathogenesis of apical periodontitis is determined by the inflammatory reactivity of periapical tissues and the magnitude of osseous destruction, both of which are modulated by the spatial distribution, structural configuration, and the pathogenic potential of microbial communities present in the root canal system [4]. Pathogenic microorganisms induce direct cytopathic effects and mediate immunomodulatory interactions with host defences through the release of bioactive molecules, including enzymatic agents, immunoglobulins, proinflammatory cytokines, chemotactic molecules, the RANK/RANKL/OPG signalling pathway, and various inflammatory mediators [5]. In response, host defense mechanisms orchestrate antimicrobial containment and microbial dissemination restriction, albeit at the expense of structural compromise to components of the apical periodontium, such as the alveolar bone, radicular cementum, and periodontal ligaments [6].

Postoperative pain management in endodontics often involves pharmaceutical strategies such as long-acting local anesthesia, prophylactic medications, postoperative analgesics, and intracanal medicaments [7]. Calcium hydroxide (Ca(OH)₂), a cornerstone intracanal medicament in endodontics since its introduction in the 1920s, is renowned for its highly alkaline nature (pH ~ 12.5). Its antimicrobial efficacy is attributed to its dissociation into calcium (Ca²⁺) and hydroxyl (OH⁻) ions. The hydroxyl ions instigate lipid peroxidation within microbial cell membranes and destabilize DNA structural integrity through helix disruption, leading to microbial cell death. Furthermore, Ca(OH)₂ depletes carbon dioxide within the root canal system, thereby suppressing the survival of CO₂-dependent bacterial species [8]. Beyond antimicrobial effects, calcium hydroxide neutralizes acidic environments, prevents tissue resorption, stimulates hard tissue formation via osteoblast/cementoblast activation, and reduces pro-inflammatory mediators (e.g., IL-1, TNF-α). It also aids periapical healing by dissolving necrotic tissue and degrading cyst linings [9].

Statins are pharmacotherapeutic agents that demonstrated anti-inflammatory, immunomodulatory, and tissue-regenerative properties, which suggest potential benefits when applied as an intracanal medicament. Previous investigations have indicated that simvastatin may exert favourable effects in endodontic and oral applications, including modulation of inflammatory mediators, promotion of periapical tissue healing, and antibacterial activity comparable to established intracanal medicaments [10]. Simvastatin has been reported to exhibit broad-spectrum antibacterial activity against clinically relevant Gram-positive and Gram-negative microorganisms [11]. Studies indicate that simvastatin interferes with multiple bacterial biosynthetic pathways and cellular functions, including inhibition of protein synthesis. Its antibacterial action has been attributed to structural modifications of teichoic acid and a reduction in surface alanine residues in Gram-positive bacteria, mechanisms that impair biofilm formation and disrupt the development of the protective polysaccharide matrix [12, 13]. Additional experimental evidence has shown that simvastatin can suppress the expression of pro-inflammatory cytokines such as interleukin-6 and interleukin-8 in oral epithelial cells [14]. The biological effects of simvastatin are primarily attributed to its role as a competitive inhibitor of 3-hydroxy-2-methyl-glutaryl coenzyme A reductase, leading to reduced cholesterol biosynthesis. Beyond lipid lowering, this mechanism is associated with pleiotropic effects, including regulation of inflammatory pathways, reduction of oxidative stress, enhancement of endothelial function, and modulation of immune responses. Moreover, simvastatin has shown promise in addressing conditions like chronic periodontitis, alveolar bone loss, dental implant osseointegration, and tissue healing, as well as potentially offering anti-cancer effects [15].

IL-6, a dual-role cytokine, regulates immune responses by promoting inflammation (via TNF-α/IL-1) and later suppressing it. It is produced by both immune and stromal cells during infection or trauma, it drives antibody production, T-cell activation, acute-phase protein synthesis, angiogenesis, and osteoclast differentiation, making it central to inflammatory cascades [16]. IL-8, a proinflammatory cytokine, recruits neutrophils to infection sites, enhancing bacterial clearance. Elevated in 95% of periapical exudates, I t correlates with acute apical inflammation and periodontal pain severity [17]. Both cytokines highlight molecular pathways targeted by simvastatin to mitigate inflammation and improve clinical outcomes in oral diseases.

Simvastatin was used previously as an intracanal medication in a study by Shroff et al. [10] that evaluated its antibacterial efficacy versus double antibiotic paste as intracanal medicaments in single-rooted teeth with apical periodontitis, with no statistically significant difference between the two groups. Previous reports in the literature have explored its effect on inflammatory mediators in an in-vitro model [18–20], or assessed its effect on periapical lesions in either animal models [21, 22] or after systemic administration [23]. To our knowledge, no prior research has evaluated the influence of simvastatin as an intracanal therapeutic agent on postoperative pain intensity or the expression of (IL-6) and (IL-8) in periapical exudates among patients diagnosed with symptomatic apical periodontitis. The present study aimed to compare the effect of using Simvastatin as an intracanal medication versus calcium hydroxide intra-canal medication on incidence and intensity of postoperative pain and periapical fluid levels of IL-6 and IL-8 one-week post-instrumentation in patients with necrotic mandibular premolars with symptomatic apical periodontitis. The null hypotheses were as follows: (1) there was no difference in postoperative pain between Simvastatin (intervention) and Ca(OH)₂ (comparator) groups when used as intracanal medications, and 2) there was no difference between the two groups in the expression of IL- 6 and IL-8 in the periapical fluids.

## Methods

### Study design and settings

The Consolidated Standards of Reporting Trials (CONSORT) 2025 guidelines were used in writing this randomized clinical trial [24]. This study was planned as a single-center, parallel, randomized trial. The study was double-blinded, with the patient, outcome assessor, and laboratory specialist blinded to group allocation. Operator blinding was maintained until treatment allocation was disclosed immediately before intracanal medicament placement, thereafter, blinding was not feasible due to the visible differences in the appearance of the intracanal medicaments. The operator (ME) performed the eligibility assessment, confirmed the diagnosis of symptomatic apical periodontitis, recorded the baseline periapical index (PAI) score, and completed all treatment procedures up to and including chemomechanical preparation and baseline periapical fluid sample collection before treatment allocation was disclosed. Following completion of these procedures, patients were randomized, after which the co-investigator (DA) revealed the assigned intervention to the operator to permit placement of the allocated intracanal medicament. Therefore, the operator became aware of treatment allocation only during intracanal medicament placement. Patient blinding was maintained throughout the study. The intracanal medicaments were placed within the root canal system under rubber dam isolation and were not visible to participants during treatment. In addition, the commercial labelling of the calcium hydroxide syringe was masked, and patients were not informed of their group allocation, or the identity of the medicament used. Patient recruitment and treatment were done in the clinic of the Department of Endodontics, Faculty of Dentistry, Cairo University, Cairo, Egypt. In the duration between September 2022 and July 2024. The study protocol and informed consent were reviewed and approved by the research ethics committee, Faculty of Dentistry, Cairo University, Egypt, on 11-9-2022, and following the Helsinki Declaration of 1975, as revised in 2000. (Approval number: 11-9-22).The trial was registered at clinicaltrials.gov (NCT 05525013) (CEBD-CU-2022-8-30). Participants received comprehensive explanations of treatment procedures and potential risks through verbal and written formats. Written informed consent, detailing study objectives and follow-up obligations, was obtained prior to enrolment, requiring adherence to protocols throughout the study period.

### Sample size calculation

Based on a previous study [25], sample size calculation was performed based on the primary outcome (postoperative pain) to be 26 patients (13 in each group) with 80% power and 5% significance level to reject the null hypothesis that the population proportions were equal. To compensate for the possible losses 15% was added so the sample size was increased to 30 patients (15 in each group). Sample size calculation was achieved using PS: Power and Sample Size Calculation Software Version 3.1.6 (Vanderbilt University, Nashville, Tennessee, USA).

### Randomization

Thirty patients were divided randomly into two groups. A random sequence of numbers (1–30) was generated via computer software (http://www.random.org/). The co-investigator (DA) generated and securely maintained the allocation list linking each study number to its assigned intervention. Immediately before intracanal medicament placement, each patient randomly selected one folded, concealed paper containing a study number form a container. The operator (ME) then contacted the co-investigator (DA), who identified the corresponding treatment allocation from the concealed allocation list and disclosed the assigned intervention, allowing placement of the intracanal medicament.

### Eligibility criteria

The inclusion criteria comprised: systemically healthy patients (ASA Class 1), females and males, aged between 20 and 50, diagnosed with symptomatic apical periodontitis secondary to pulp necrosis in single-rooted mandibular premolars; with radiographic confirmation of periapical radiolucency (Periapical index (PAI) 3) [26] and moderate to severe preoperative pain on the numerical rating scale (NRS) [27].

The exclusion criteria comprised: medically compromised patients (ASA Class 2 and 3), pregnant females, patients using analgesics or antibiotics within 2 weeks before treatment which may affect their pain or interleukin levels, patients with bruxism or parafunctional clenching habits, to prevent mechanical exacerbation of inflammation in the affected tooth, non-restorable or periodontally affected teeth, retreatment cases, presence of greater than grade I mobility, calcification, resorption, swelling or sinus tract.

### Diagnosis

The following data were recorded on a specific form for each patient: name, phone number, address, medical, dental, and chief complaint history. The selected patients presented with pain on biting involving mandibular premolars. After thorough extra-, intra-oral clinical examination and digital periapical radiograph of the suspected tooth, the diagnostic confirmation of pulp necrosis with symptomatic apical periodontitis was established through the following criteria: (1) patient-reported history of pain during mastication, (2) absence of pulpal responsiveness to standardized sensibility testing, (3) positive findings on controlled percussion assessment (using the contralateral tooth as a comparative baseline), and (4) radiographic evidence of single-rooted teeth exhibiting periapical radiolucency not exceeding 2 mm in diameter. Patients recorded; on NRS chart; their pre-operative pain level.

### Clinical procedures

The endodontic treatment was completed in two visits separated by one week. The same operator (ME) performed all procedures. Both groups received the same procedures for chemo-mechanical preparation. The tooth was anaesthetized using infiltration technique with 1.8 ml of 4% Articaine with 1:100,000 epinephrine (ARTINIBSA; Inibsa lab., Barcelona, Spain). The tooth, rubber dam clamp, and surrounding isolation field were disinfected sequentially with 30% hydrogen peroxide (H₂O₂) followed by 5% sodium hypochlorite (NaOCl). Residual disinfectants were neutralized using 5% sodium thiosulfate. Under magnification via a dental operating microscope (OPMI PICO; Carl Zeiss, Oberkochen, Germany), access cavity preparation was initiated with a high-speed handpiece utilizing a round bur and Endo-Z bur (Dentsply Maillefer, Ballaigues, Switzerland). Following access refinement, the operative field and pulp chamber underwent disinfection using the aforementioned protocol. A single root canal configuration was verified, and canal patency was established using #10 and #15 K-type hand files (MANI, INC., Japan), sized according to the initial apical foramen diameter. Working length (WL) determination was achieved by advancing a #15 K-file to the apical constriction, as identified electronically via a Root ZX apex locator (J Morita Corp, Japan), and radiographically confirmed to terminate 0.5 mm coronal to the radiographic apex. Root canal instrumentation was performed using nickel-titanium ProTaper Next rotary files (Dentsply Maillefer, Ballaigues, Switzerland) in an X-Smart endodontic motor, adhering to the manufacturer’s protocol, with preparation finalized to X4 file (size #40, taper 0.06). During instrumentation, each file was coated with ethylenediaminetetraacetic acid (EDTA) gel (Meta Biomed Co. Ltd, Korea) prior to canal insertion. Irrigation protocols included sequential delivery of 2.5% sodium hypochlorite (NaOCl) via a 30-gauge side-vented needle (Steri irrigation tips; Diadent, Korea) attached to a 5 mL syringe, positioned 2 mm short of the working length. A standardized total of 25 mL NaOCl was administered in 5 mL aliquots (pre-instrumentation, post-each file, and post-X4 completion), followed by a final 1-minute rinse with 17% EDTA (1 mL). Intermediate saline irrigation was implemented to prevent chemical interaction between NaOCl and EDTA. Prior to sampling, the root canal was thoroughly dried using sterile paper points to avoid contamination with blood or irrigant. Dryness was achieved using sterile #40 paper points (Meta Biomed Co. Ltd, Korea). Post-instrumentation microbial sampling (PS-1) involved aseptically placing three sterile size 20 paper points 2 mm beyond the apical foramen for 1 min each, as per [28]. This procedure was repeated three times per canal. Collected paper points were stored in sterile microcentrifuge tubes (Merck, USA) containing 2 mL of normal saline and cryopreserved at − 80 °C pending analysis.

Immediately before intracanal placement, patients were randomly assigned to one of the two groups (*n* = 15) as follows:

#### Simvastatin group

Methylcellulose gel (MC) served as the simvastatin delivery vehicle in this study. 100 ml of distilled water were boiled to create a 3% MC gel followed by adding Sodium carboxymethyl cellulose (NA- CMC) by sprinkling with continuous stirring till gel formation. The gel was allowed to cool at room temperature for 24 h. Subsequently, the simvastatin powder was precisely measured, dissolved in absolute ethanol, incorporated into the pre-prepared methylcellulose (MC) gel under continuous stirring until a visually homogeneous formulation was obtained. To produce 0.5% simvastatin gel, 20 mg of simvastatin powder was dissolved in 1 ml absolute ethanol, then added to 19 gm of MC gel. All preparation steps were performed in the (Drug manufacturing Unit) at the Faculty of Pharmacy, according to methods reported in previous studies [20, 29]. For intracanal application, the simvastatin gel was delivered using a disposable intracanal tip positioned 1–2 mm short of the working length. A standardized application protocol was followed for all cases to ensure consistent placement of the medicament within the canal system, however, the exact quantity of medicament delivered into each canal was not directly quantified.

#### Calcium hydroxide group

Calcium hydroxide paste (Metapaste, Meta Biomed Co., Ltd, Korea) was delivered into the canal via a disposable applicator tip, positioned 1–2 mm short of the working length. The access cavity was sealed with a sterile cotton pellet and a glass ionomer interim restoration (Meta Biomed Co. Ltd, Korea). Participants were instructed to document postoperative pain intensity using a numeric rating scale (NRS) at 6, 12, 24, and 48 h, followed by a one week recall visit. At the recall appointment, rubber dam isolation and preoperative disinfection protocols were reinstated. Intracanal medicaments were removed using 5 mL of 17% EDTA, followed by copious irrigation with saline. A pre-obturation microbial sample (PS-2) was collected using standardized protocols. Final irrigation with 2.5% NaOCl and 17% EDTA was performed in all cases. Master gutta-percha cones (X4; Dentsply-Maillefer, Switzerland), matching the final preparation dimensions, were fitted to working length and radiographically verified. Following canal drying with paper points, obturation was completed via a modified single-cone technique using a bioceramic sealer (CeraSeal, Meta Biomed Co. Ltd, Korea) in conjunction with X4 and auxiliary gutta-percha cones (Meta Biomed Co. Ltd, Korea). The access cavity was permanently restored with composite resin (Meta Biomed Co. Ltd, Korea), with occlusal contacts adjusted. Participants again recorded postoperative pain via NRS at 6, 12, 24, and 48 h, and were contacted by telephone for reminding and ensuring accurate readings.

### Postoperative pain intensity assessment (Primary outcome)

Patients were clearly instructed on how to use the Numerical Rating Scale (NRS) for accurately reporting their pain levels.; which comprises 11 points from 0 to 10; where 0 = No-Pain, and 10 = Severe-Pain. They were asked to record their pre- and post-operative pain levels; at 6, 12, 24 h, and 48 h post-instrumentation and post-obturation; and submit it to the principal investigator both in real-time (by phone) and in writing (at the second appointment). Patients were instructed to contact the main investigator in case of any flare-ups and were prescribed Ibuprofen 400 mg (Novartis Pharma) as an analgesic. Patients who required analgesics were excluded from the study to avoid more confounders. No patient had a flare-up or required prescription of analgesics in both groups.

### Cytokines (IL-6, IL-8) analysis (Secondary outcome)

After all samples were collected, IL-6 and IL-8 levels were assessed using an ELISA kit (Bioassay Technology, Zhejiang, China) with Cat. No E0090Hu and Cat. No E0089Hu, respectively. The kits were used in compliance with the manufacturer’s guidelines. To guarantee thorough mixing, 200 µl (µL) of phosphate-buffered-saline were added to the samples. These were centrifuged for 5 min at 10,000 rpm. On plates pre-coated with human IL-6 or IL-8 antibodies, the collected apical supernatants were added and bound to it. The sample’s IL-6 and IL-8 were bound by addition of biotinylated human IL-6 and IL-8 antibody, respectively. Next, the biotinylated human IL-6 and IL-8 were coupled to streptavidin-HRP (s-HRP). In a washing step; following incubation; washout of unbound s-HRP occurred. Upon adding the substrate solution, the resulting color intensity correlated linearly with the concentration of human IL-6 or IL-8. To terminate the reaction, an acidic-stop solution was added. Using spectrophotometer at 450 nm, absorbance was measured. Based on the readings obtained, concentration of IL-6 and IL-8 were determined in ng/L.

### Statistical analysis

Data were compiled and organized for analysis. Statistical evaluations were conducted using IBM SPSS Statistics software (Version 25.0; IBM Corp., Armonk, NY). Normality of data distribution was assessed via the Shapiro-Wilk test. Continuous variables were summarized descriptively as mean ± standard deviation (SD), median, and range (minimum to maximum values). Normally distributed continuous data were analyzed with independent samples t-test (age) and for non-normally distributed data, non-parametric tests were employed: the Mann-Whitney U test compared independent groups, (comparison of NRS scores at different observation periods between groups - comparison of IL-6 and IL-8 concentrations at different observation periods between groups) while Friedman test and Dunn’s post hoc test were used for intragroup difference over time. The Wilcoxon signed rank test was used for comparison of the change in IL-6 and IL-8 concentrations within each group. Categorical data (Gender and Tooth type) were expressed as frequency counts (N) and percentages (%), with intergroup differences evaluated using the chi-square test. A significance level of *p* ≤ 0.05 was adopted for all analyses.

## Results

**Fig. 1:**
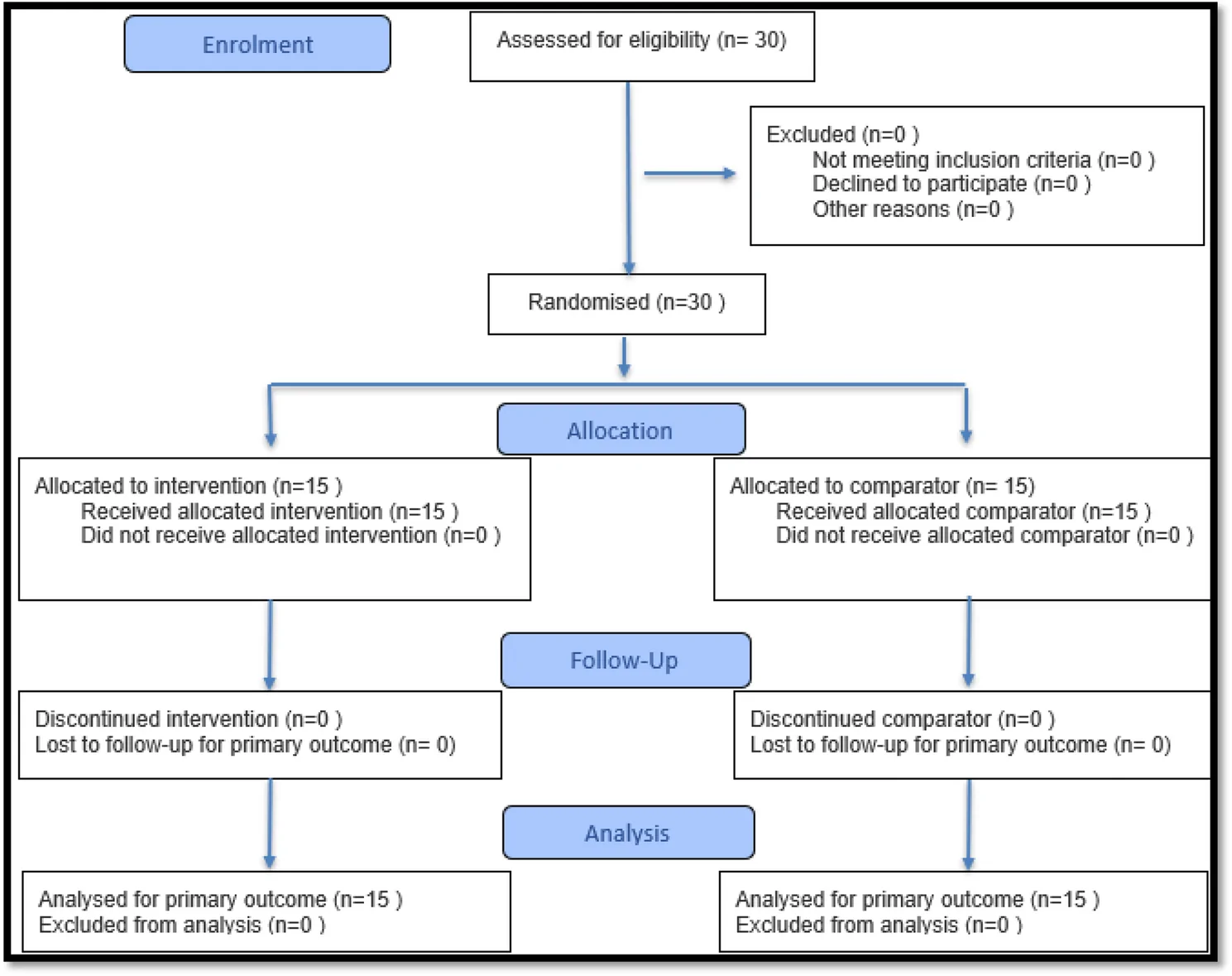
CONSORT 2025 flowchart

Statistical analysis included thirty patients. The enrolment procedure for patients and their progress through every phase of the study are shown in the CONSORT 2025 Flowchart (Fig. 1).

No patients were excluded from analysis in this study. For demographic data, descriptive statistics are presented in (Table 1).

**Table 1:** Descriptive statistics and the results of independent *t-test* for comparison of age and Chi-square test for comparison of gender and tooth type distribution between the two groups

		Simvastatin	Ca(OH)_2_	*p*-value
Age	Mean (SD)	36.5 (7.9)	33.7 (7.1)	0.289
	Median (Range)	37 (21–49)	34 (21–47)	
Gender		N (%)	N (%)	0.464
	Males Females	6 (40%) 9 (60%)	8 (53.3%) 7 (46.7%)	
		N (%)	N (%)	
Tooth type	1st premolars	4 (26.7%)	8 (53.3%)	0.221
	2nd premolars	11 (73.3%)	7 (46.7%)	

*Significant at the 0.05 level

Simvastatin showed significantly lower post-instrumentation pain intensity than calcium hydroxide (Ca(OH)₂) at 6 h (*p* = 0.037), with no significant differences at later intervals (Table 2). In the Simvastatin group, pain reduction became significant at 12, 24, and 48 h compared to preoperative levels (*p <* 0.001). For Ca(OH)₂, pain decreased significantly only by 24 and 48 h (*p <* 0.001). Both groups exhibited stabilized pain levels after 24 h, with no significant difference at 48 h (Table 2).

**Table 2:** The mean, standard deviation values and the results of Mann-Whitney U test for comparison of NRS scores at different observation periods post-instrumentation between the two groups, and the results of Friedman test and Dunn’s post hoc test for comparison of the change in post-instrumentation pain intensity with time within each group

Observation period			Simvastatin	Ca(OH)_2_	*p*-value	Effect size with 95%CI***
Preoperative		Mean (SD)	5.7^a^ (1.2)	6.8^a^ (2.4)	0.345	-1 (-3, 1)
		Median	6	6		
		(Range)	(4–8)	(4–10)		
Following 1st visit	6 h postoperatively	Mean (SD)	3.3^ab^ (1.7)	5.3^ab^ (2.9)	0.037*	-2 (-4, 0)
		Median	3	6		
		(Range)	(1–7)	(0–10)		
	12 h postoperatively	Mean (SD)	2.9^b^ (2)	4.1^ab^ (3.1)	0.267	-1 (-3, 1)
		Median	2	4		
		(Range)	(0–6)	(0–10)		
	24 h postoperatively	Mean (SD)	2.2^b^ (3.4)	3.1^bc^ (3.1)	0.325	0 (-3**,** 0)
		Median	0	2		
		(Range)	(0–10)	(0–9)		
	48 h postoperatively	Mean (SD)	1.6^b^ (3.5)	1.9^c^ (2.7)	0.595	0 (-1, 0)
		Median	0	0		
		(Range)	(0–10)	(0–7)		
p-value			< 0.001*	< 0.001*		

*Significant at the 0.05 level

**Different lower-case letters indicate statistical significance within the same column

***Hodges-Lehman’s median difference

Post-obturation pain intensity showed no significant differences between Simvastatin and Ca(OH)₂ groups at any time interval (Table 3). In the Simvastatin group, pain intensity remained stable between 6 and 12 h but significantly decreased by 24 and 48 h compared to baseline (6 h) (*p <* 0.001). Pain stabilized after 12 h, with no further changes between 12 and 48 h. For Ca(OH)₂, pain levels remained statistically similar across all intervals (6–48 h), peaking at 12 h with no significant difference (Table 3).

**Table 3:** The mean, standard deviation values and the results of Mann-Whitney U test for comparison of NRS scores at different observation periods post-obturation between the two groups, and the results of Friedman test and Dunn’s post hoc test for comparison of the change in post-obturation pain intensity with time within each group

Observation period			Simvastatin	Ca(OH)_2_	*p*-value	Effect size with 95%CI***
Following 2nd visit	6 h postoperatively	Mean (SD)	1.5^a^ (1.4)	0.9 (1.7)	0.187	1 (0**,** 2)
		Median	1	0		
		(Range)	(0–4)	(0–5)		
	12 h postoperatively	Mean (SD)	0.7^ab^ (1.2)	1.1 (2.0)	0.870	0 (0**,** 0)
		Median	0	0		
		(Range)	(0–4)	(0–5)		
	24 h postoperatively	Mean (SD)	0.3^b^ (0.7)	0.9 (1.8)	0.870	0 (0**,** 0)
		Median	0	0		
		(Range)	(0–2)	(0–5)		
	48 h postoperatively	Mean (SD)	0.2^b^ (0.6)	0.7 (1.8)	0.935	0 (0, 0)
		Median	0	0		
		(Range)	(0–2)	(0–6)		
*p*-value			< 0.001*	0.682		

*Significant at the 0.05 level

**Different lower-case letters indicate statistical significance within the same column

***Hodges-Lehman’s median difference

**Fig. 2:**
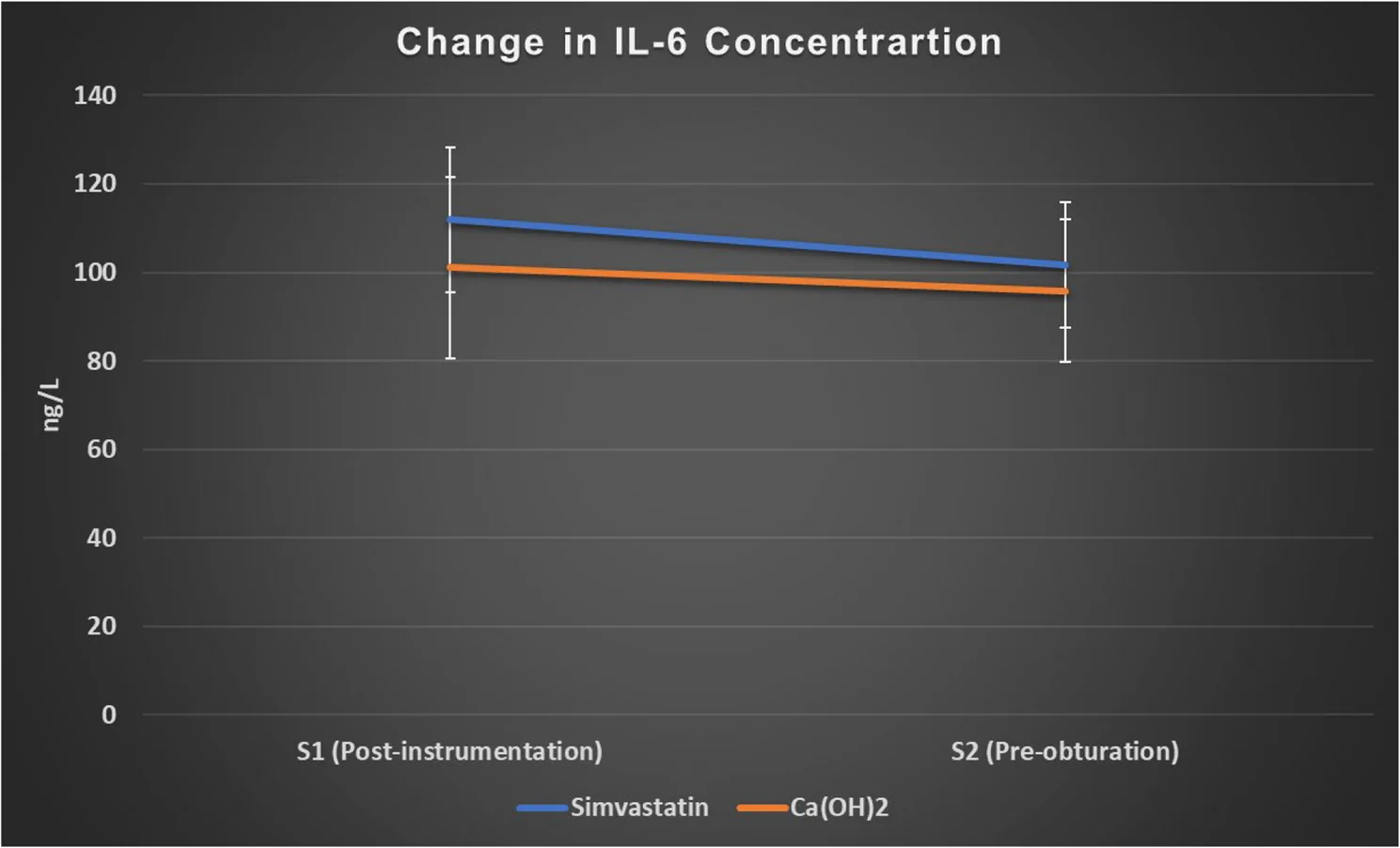
line chart representing the change in IL-6 concentration within each group

**Fig. 3:**
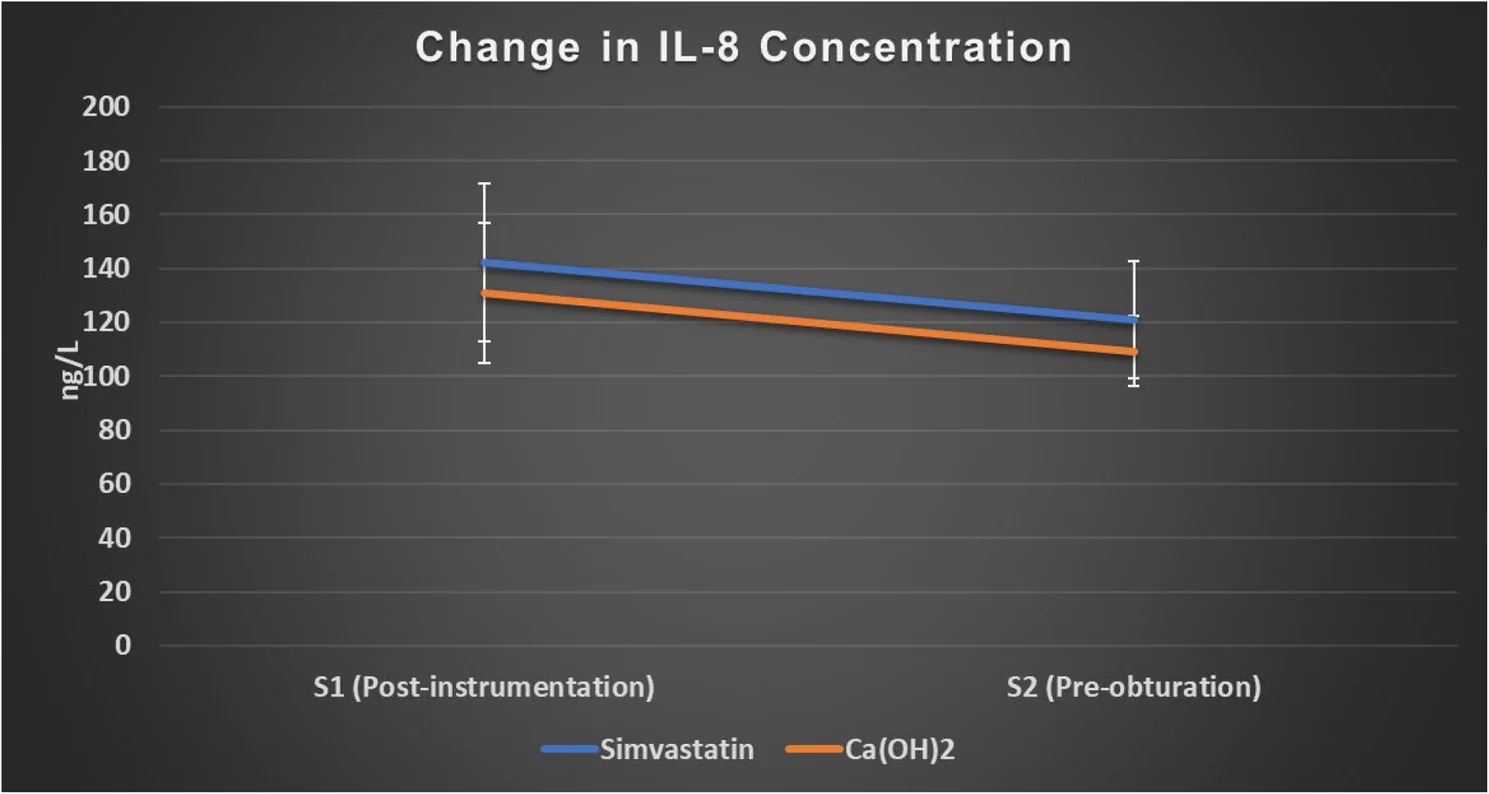
line chart representing the change in IL-8 concentration within each group

There was no statistically significant difference between postoperative levels of IL-6 and IL-8 in both groups (Tables 4 and 5). However, Simvastatin group showed a statistically significant reduction in both IL-6, (*p* = 0.017) and IL- 8, (*p* = 0.002) levels compared with their preoperative levels, while Ca(OH)2 group showed a statistically significant reduction in IL-8 levels only, (*p* = 0.005), (Figs. 2 and 3).

**Table 4:** Mean, standard deviation values and the results of Mann-Whitney U test for comparison of IL-6 concentration between the two groups at the intervals (PS-1,PS-2), and the results of Wilcoxon signed rank test for comparison of the change in IL-6 concentration within each group

		Simvastatin	Ca(OH)_2_	*p*-value	Effect size with 95%CI**
PS-1	Mean (SD)	111.9 ± 16.4	101.1 ± 20.5	0.067	13.5 (-1**,** 26.3)
	Median (Range)	109.5 (74.5–137.4)	99.1 (78.2–145.3)		
PS-2	Mean (SD)	101.8 ± 14.2	95.9 ± 16.2	0.367	4.2 (-6**,** 16.6)
	Median (Range)	102.8 (74.2–134.9)	98.6 (68.8–122.8)		
p-value		0.017*	0.211		
Effect size with 95%CI**		-11.9 (-17.9, -2.5)	-5.7 (-14.3, 3.4)		

*Significant at the 0.05 level

**Hodges-Lehman’s median difference

**Table 5:** Mean, standard deviation values and the results of Mann-Whitney U test for comparison of IL-8 concentration between the two groups at the intervals (PS-1,PS-2), and the results of Wilcoxon signed rank test for comparison of the change in IL-8 concentration within each group

		Simvastatin	Ca(OH)_2_	*p*-value	Effect size with 95%CI**
PS-1	Mean (SD)	142.2 ± 29.5	130.9 ± 26.0	0.250	9.5 (-9.4**,** 33.4)
	Median (Range)	137.1 (99.2–196.5)	121.7 (102.2–201.8)		
PS-2	Mean (SD)	121.1 ± 21.7	109.3 ± 13.0	0.174	11.3 (-2**,** 23.5)
	Median (Range)	122.4 (93.2–175.3)	103.6 (96.5–145.7)		
p-value		0.002*	0.005*		
Effect size with 95%CI**		-21.3 (-31.1**,** -8.4)	-17.7 (-33.1**,** -6.2)		

*Significant at the 0.05 level

** Hodges-Lehman’s median difference

## Discussion

The first null hypothesis was rejected, as there was a significant difference in postoperative pain values between Simvastatin and Ca(OH)₂ groups at 6-hours post-instrumentation. While the second null hypothesis was accepted as there was no difference between the two groups in the expression of IL-6 and IL-8 in the periapical fluids.

Postoperative pain etiology, though incompletely understood, is multifactorial, involving microbiological, mechanical, and chemical interactions. Risk factors include variables such as anxiety, sex, age, preoperative pulpal/periapical pathology, tooth type, history of pain or percussion sensitivity, irrigant selection, and single vs. multiple visit treatment [30].

Emerging evidence implicates (IL-6) in the pathological progression of human periapical diseases, with empirical studies showing a positive correlation between IL-6 concentrations and periapical lesion dimensions. Neutrophils and macrophages within these lesions exhibit upregulated IL-6 expression following bacterial antigenic stimulation. Furthermore, clinically symptomatic, expansive, and epithelialized lesions demonstrate quantitatively elevated IL-6 levels relative to asymptomatic or smaller counterparts [16]. Similarly, (IL-8) has been identified as a biomarker of symptomatic apical periodontitis, with studies documenting its increased expression in symptomatic clinical presentations [31, 32].

Simvastatin reduces (IL-6) release in mononuclear cells and suppresses (IL-6) and (IL-8) production in oral epithelial cells [14, 33]. It is effective in managing chronic periodontitis through local or systemic use and benefits dental health by aiding bone regeneration, tissue repair, implant osseointegration, and reducing inflammation [15].

The study involved participants with single-canal necrotic teeth exhibiting minimal periapical radiolucency (≤ 2 mm), selected to standardize anatomical complexity and inflammatory mediator sampling while avoiding variability from multi-rooted teeth. Mandibular premolars were prioritized due to their single-rooted anatomy and higher postoperative pain risk linked to the mandible’s dense bone structure [34]. Patients with preoperative pain—a known predictor of post-treatment pain—were included [35], while those with large radioluciencies (indicative of chronic infection or cystic changes) were excluded due to their adverse impact on the success rate of the treatment [36].

Endodontic treatment was conducted over two visits to allow quantification of IL-6 and IL-8 levels in periapical fluid following canal instrumentation and intracanal medicament placement. Periapical fluid sampling was selected in preference to gingival crevicular fluid to minimize potential confounding from periodontal inflammation [37]. A one-week inter-appointment period was selected because it represents a commonly used protocol for intracanal medication in clinical endodontic studies and allows adequate time for the medicament to exert its biological effects before obturation [9, 38–40].

In the present study, a 0.5 mg dose of simvastatin was used. Evidence suggests that the therapeutic effects of simvastatin are dose-dependent, and preclinical studies indicate that a 0.5 mg dose is effective in suppressing inflammatory pathways and promoting osteogenesis at sites of bone resorption. Importantly, these studies, which investigated local administration, report a favourable safety profile relevant to site-specific applications, supporting the use of this dose in intracanal or dental contexts [14, 41].

Pain assessment was conducted preoperatively and at 6, 12, 24, and 48 h postoperatively, following the first and second visits. Assessments commenced at the 6-hour mark to confirm that the effects of the local anaesthetic agent had completely dissipated [42]. The selected follow-up periods were as specified because periapical inflammation resulting from endodontic treatment activates proprioceptive nerve fibers within the periodontal ligament, leading to postoperative discomfort that typically diminishes to minimal levels within 24 to 48 h [43].

The selection of sample collection time intervals as post-instrumentation and pre-obturation (one week later) phases to quantify reductions in IL-6 and IL-8 levels within periapical fluid was informed by clinical evidence demonstrating significant changes in inflammatory mediators’ levels between initial and follow-up visits [44].

Intracanal interappointment dressings are a recommended strategy to minimize postoperative pain and flare- ups in necrotic teeth. Studies indicate that intracanal steroids, anti-inflammatory agents, antibiotics, or corticosteroid- antibiotic combinations are associated with lower postoperative pain levels compared to calcium hydroxide or no medicament placement post-debridement [45]. This comes in agreement with the current study. The lower postoperative pain observed at 6 h with simvastatin compared to calcium hydroxide may be attributed to its pleiotropic molecular effects at the periapical site. Simvastatin inhibits leukocyte-endothelial adhesion by downregulating ICAM-1, VCAM-1, and E-/P-selectin expression and suppressing the Rho/ROCK pathway, thereby limiting neutrophil and monocyte infiltration into inflamed periapical tissues. Reduced leukocyte recruitment results in decreased local production of proinflammatory cytokines, including IL-1β, IL-6, and TNF-α, which sensitize nociceptive fibers. Additionally, simvastatin suppresses COX-2 and prostaglandin E2 synthesis and may modulate TRPV1 and Nav1.7 channels on peripheral nociceptors, collectively contributing to faster pain relief. Its antioxidant and endothelial-stabilizing effects further reduce tissue edema, decreasing mechanical stimulation of pain fibers [46, 47]. It was demonstrated by Shroff et al. [10] that simvastatin had comparable antibacterial effect to double antibiotic paste when used as intracanal medicaments in single-rooted teeth with apical periodontitis with no statistically significant intergroup disparity in bacterial load reduction. Although the difference in NRS scores at 6 h post-instrumentation between the simvastatin and calcium hydroxide groups was statistically significant, this magnitude aligns with the minimal clinically important difference (MCID) for acute pain on the NRS, which is generally considered to be 1.5–2 points [48]. Therefore, the observed reduction may represent a clinically perceptible early analgesic effect of simvastatin. However, this difference was observed only at the 6-hour time point and was not consistently across subsequent postoperative assessments. Furthermore, because postoperative pain was evaluated at multiple prespecified time points, repeated statistical comparisons may increase the risk of Type I error. Although formal adjustments such as the Bonferroni correction may be overly conservative for correlated repeated-measures data, particularly when analyses are prespecified, this isolated finding should nevertheless be interpreted cautiously within the context of the overall pattern of postoperative pain outcomes and confirmed in future studies.

Regarding the postoperative IL-6 levels in periapical fluids, there was no statistically significant difference between postoperative IL-6 levels in Simvastatin and calcium hydroxide groups. However, the Simvastatin group exhibited a statistically significant decrease in IL-6 levels between post-instrumentation and pre-obturation sampling phases. The reduction in IL-6 observed in the present study is consistent with the anti-inflammatory properties of simvastatin reported in previous investigations. Sharma et al. [20] demonstrated decreased IL-6 production when simvastatin was incorporated into root canal sealers in an in-vitro fibroblast cell culture model. However, direct comparison with the present findings should be interpreted cautiously because that study evaluated cellular responses under controlled laboratory conditions, whereas the present investigation assessed clinical inflammatory responses within the complex periapical environment. More recently, Harikrishnan et al. [39] reported significant modulation of inflammatory mediators following the use of simvastatin as an intracanal medicament, including reduced IL-6 and MMP-9 levels and increased IL-10 expression, together with favourable effects on interappointment pain. These findings provide clinical support for the anti-inflammatory effects of intracanal simvastatin and are consistent with the results of the present study. Nevertheless, some differences between the two studies should be considered. Harikrishnan et al. used a 1.2% simvastatin formulation, whereas the present study employed a 0.5% concentration. As the biological effects of simvastatin may be concentration-dependent, differences may influence the magnitude of the observed biomarker changes between studies. In contrast, the calcium hydroxide Ca(OH)₂ group showed no statistically significant difference in IL-6 concentrations across the same intervals. This may be attributed to duration of calcium hydroxide placement. The transient efficacy of calcium hydroxide as an intracanal medicament stems from its rapid dissociation into calcium and hydroxyl ions. Hydroxyl ions diffuse through dentinal tubules within 1 h of placement, temporarily elevating the periradicular pH to ~ 11.1, which disrupts proinflammatory cytokines such as IL-8 [9]. However, extracellular fluid buffers maintain physiological pH (7.35–7.45), neutralizing hydroxyl ions and limiting sustained pH elevation [49]. This buffering effect prevents prolonged cytokine denaturation, explaining the minimal reduction in periradicular IL-6 levels.

Regarding the postoperative IL-8 levels in periapical fluids, postoperative IL-8 levels were statistically comparable between both treatment groups. Moreover, the Simvastatin group demonstrated a statistically significant reduction in IL-8 concentrations from post-instrumentation to pre-obturation sampling. This comes in accordance with several in-vitro studies and studies assessing the effect of systemically administered Simvastatin [14, 50–52]. To the best of our knowledge, no previous clinical study has specifically investigated the effect of intracanal simvastatin on IL-8 expression, making this aspect of the present study novel. Similarly, the calcium hydroxide Ca(OH)₂ group also exhibited a significant decline in IL-8 levels over the same period. This result is in an agreement with reported evidence of effect of calcium hydroxide intracanal medication on various inflammatory mediators [53–56].

To our knowledge, this clinical trial is among the first to evaluate 0.5% simvastatin as an intracanal medicament and the first to investigate its effects on both IL-6 and IL-8 levels in periapical fluid. The effects of statins differ according to the route of administration, with systemic delivery producing primarily metabolic and systemic immunomodulatory effects, whereas local application allows higher site-specific concentrations that may enhance anti-inflammatory, antimicrobial, and regenerative actions. In endodontic therapy, local simvastatin delivery may therefore provide more targeted modulation of periapical inflammation than systemic administration.

Several limitations should be considered when interpreting the findings of the present study. First, postoperative pain is inherently subjective and may be influenced by individual differences in pain perception, preoperative anxiety, tissue trauma induced by local anesthetic administration, and procedural factors associated with treatment. Although standardized clinical procedures were followed for all participants, these variables cannot be eliminated. Second, the sample size calculation was based on the primary outcome (postoperative pain), and no separate biomarker-specific power calculation was performed for IL-6 and IL-8. Therefore, the study may not have been specifically powered to detect smaller differences in cytokine levels between groups. Accordingly, the cytokine findings should be interpreted with appropriate caution. Furthermore, although the concentration of simvastatin within the gel formulation was standardized, the exact quantity of medicament delivered into each canal was not directly quantified, and minor variations related to differences in canal anatomy may have occurred. In addition, intracanal medicaments were evaluated over a single one-week inter-appointment period. While this duration reflects common clinical practice and was selected to standardize treatment conditions, longer application periods may influence the antimicrobial and anti-inflammatory effects of the medicaments, particularly calcium hydroxide. Finally, the single-center design may limit the generalizability of the findings. Future multicenter studies with larger sample sizes, biomarker-specific power calculations, and longer follow-up periods are warranted to confirm these findings and further evaluate the effects of intracanal simvastatin across different concentrations, delivery systems, inflammatory mediators, clinical conditions, and long-term healing outcomes.

## Conclusion

Within the limitations of this study, it can be concluded that Simvastatin used as an intracanal medication, demonstrated clinical and biological outcomes comparable to calcium hydroxide in treating necrotic teeth with symptomatic apical periodontitis. The use of Simvastatin showed lower post-instrumentation pain intensity score at 6 h postoperatively compared with calcium hydroxide, however, this finding should be interpreted with caution within the context of the overall pain outcomes. Simvastatin showed an equally favorable periapical tissue response to calcium hydroxide with no significant difference in IL-6 and IL-8 levels in periapical fluids. However, Simvastatin group showed a statistically significant reduction in both IL-6 and IL-8 levels compared with their preoperative levels, while Ca(OH)2 group showed a statistically significant reduction in IL-8 levels only.

## Data Availability

The datasets generated and/or analysed during the current study are not publicly available due to (The authors’ university hospital has a specific policy governing data sharing that mandates controlled access rather than open publication for raw data. Also, participants consented only to the specific study described, not to unrestricted public deposition of their raw data) but are available from the corresponding author on reasonable request.
